# A Previously Undiscovered Circular RNA, circTNFAIP3, and Its Role in Coronavirus Replication

**DOI:** 10.1128/mBio.02984-21

**Published:** 2021-11-16

**Authors:** Liuyang Du, Xingbo Wang, Junli Liu, Jiarong Li, Shengnan Wang, Jing Lei, Jiyong Zhou, Jinyan Gu

**Affiliations:** a Institute of Immunology, College of Veterinary Medicine, Nanjing Agricultural University, Nanjing, China; b MOA Key Laboratory of Animal Virology, Zhejiang University Center for Veterinary Sciences, Hangzhou, China; c Collaborative Innovation Center and State Key laboratory for Diagnosis and Treatment of Infectious Diseases, The First Affiliated Hospital, Zhejiang University, Hangzhou, China; Virginia Polytechnic Institute and State University

**Keywords:** deltacoronavirus, circular RNA, circTNFAIP3, virus replication, apoptosis

## Abstract

Circular RNAs (circRNAs) are a newly discovered class of noncoding RNAs (ncRNAs) present in various tissues and cells. However, the functions of most circRNAs have not been verified experimentally. Here, using deltacoronavirus as a model, differentially expressed circRNAs in cells with or without deltacoronavirus infection were analyzed by RNA sequencing to characterize the cellular responses to RNA virus infection. More than 57,000 circRNA candidates were detected, and seven significantly dysregulated circRNAs were quantitated by real-time reverse transcription-PCR. We discovered a previously unidentified circRNA derived from the *TNFAIP3* gene, named circTNFAIP3, which is distributed and expressed widely in various tissues. RNA viruses, including deltacoronaviruses, rather than DNA viruses tend to activate the expression of endogenous circTNFAIP3. Overexpression of circTNFAIP3 promoted deltacoronavirus replication by reducing the apoptosis, while silencing of circTNFAIP3 inhibited deltacoronavirus replication by enhancing the apoptosis. In summary, our work provides useful circRNA-related information to facilitate investigation of the underlying mechanism of deltacoronavirus infection and identifies a novel circTNFAIP3 that promotes deltacoronavirus replication via regulating apoptosis.

## INTRODUCTION

Circular RNAs (circRNAs), covalently closed loop structures with neither 5′-to-3′ polarity nor a polyadenylated tail, are a novel type of endogenous noncoding RNAs (ncRNAs) that are abundant in the eukaryotic transcriptome (1, 2). With the help of high-throughput sequencing and computational approaches, numerous circRNAs have been identified in various cell lines and tissues (1, 3–5). CircRNAs were previously considered the functionless products of missplicing or splicing errors (6). However, recent studies showed that circRNAs may play multiple regulatory roles in biological and pathological processes including gene regulation (7), alternative splicing (8), cell growth regulation (9), microRNA (miRNA) sponges (10), neural development (11), and carcinogenesis (12, 13). Some DNA tumor viruses, such as Epstein-Barr virus and Kaposi’s sarcoma herpesvirus, reportedly encode circRNAs (14, 15). In addition, some RNA sequencing (RNA-seq) reports have described host circRNAs in virus-infected cells or tissues, including those harboring herpes simplex virus 1 (HSV-1) (16), transmissible gastroenteritis virus (17), and epidemic diarrhea virus (18). However, characterization and functional analysis of these virus/host-encoded circRNAs appear to be limited.

*Coronavirus* (CoV), belonging to the family *Coronaviridae*, is a single-strand positive-sense RNA virus with an envelope. CoVs are separated into four genera based on phylogeny: *Alphacoronavirus*, *Betacoronavirus*, *Gammacoronavirus*, and *Deltacoronavirus* (https://talk.ictvonline.org/taxonomy/). Porcine deltacoronavirus (PDCoV), discovered in pig feces in Hong Kong in 2012 (19), causes severe diarrhea, dehydration, and vomiting in nursing piglets (20). Deltacoronaviruses have been identified in many songbird species and in leopard cats (19), but PDCoV is the only one that has been cultured *in vitro*, making it a perfect model for studying deltacoronaviruses. The PDCoV genome has a short 5′ untranslated region (5′-UTR) and 3′-UTR and is ∼25.4 kb in length. It encodes structural spike (S), envelope (E), membrane (M) and nucleocapsid (N) proteins, as well as 15 nonstructural proteins (21). After the first outbreak in Ohio in 2014, PDCoV was subsequently identified in diseased pig farms in other parts of the United States (22) and many Asian countries (23–25), representing a considerable threat to the swine industry. PDCoV has been successfully isolated and propagated in LLC porcine kidney (LLC-PK) cells and swine testicular (ST) cells (26), and PDCoV infection can induce apoptosis in these two cell lines (27).

Viruses subvert macromolecular pathways in infected host cells to facilitate viral gene amplification or to counteract innate immune responses. It has been reported that the PDCoV proteins Nsp5, NS6, and N inhibit the production of beta interferon (IFN-β) and thereby antagonize the innate immune response of the host (28–30). Accumulating evidence indicates that many endogenous transcripts, especially ncRNAs, may play an important role in the struggle between hosts and viruses (31–33), but little is known about the role of host ncRNAs in deltacoronavirus infection.

In this study, using PDCoV as a model deltacoronavirus, we identified 57,704 circRNA candidates in deltacoronavirus-infected and mock-infected cells using RNA-seq and confirmed seven significantly dysregulated circRNAs. We discovered the circRNA TNFAIP3 (circTNFAIP3) derived from the *TNFAIP3* gene. Upregulating circTNFAIP3 expression restrained the cleavage of caspase-3 and promoted deltacoronavirus replication, whereas silencing circTNFAIP3 had the opposite effect, indicating that circTNFAIP3 is a positive regulator of deltacoronavirus replication by inhibiting apoptosis. Given the high stability of circRNAs, our findings may provide a potential antiviral target for coronavirus infection strategies.

## RESULTS

### RNA-seq and circRNA profiling of deltacoronavirus-infected cells.

**FIG 1:**
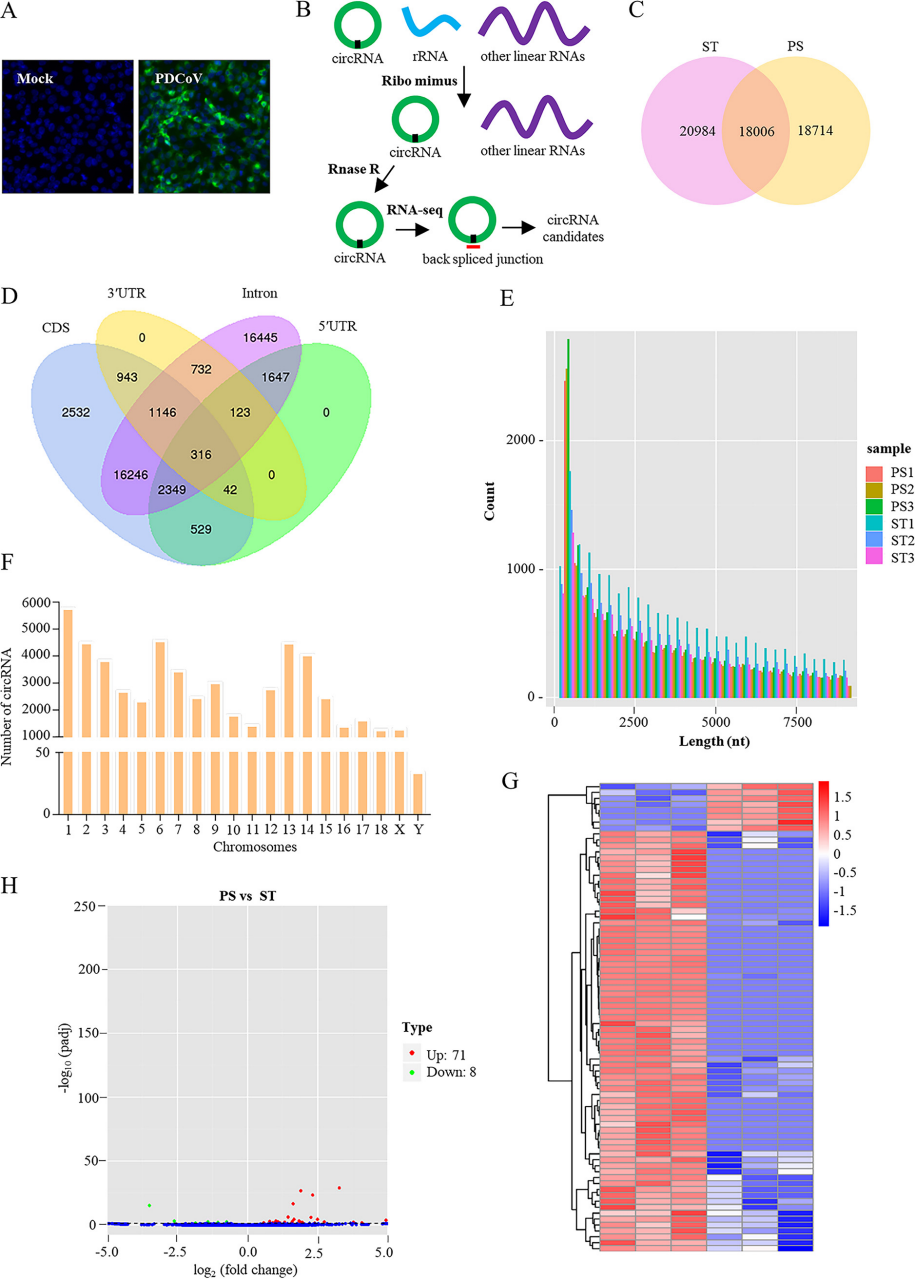
RNA-seq and circRNA profiling of mock- and PDCoV-infected cells. (A) IFA image of mock- and PDCoV-infected ST cells. Cells were infected with or without PDCoV at an MOI of 10 for 11 h and then stained with anti-PDCoV S protein polyclonal antibody (green). Nucleic acids were labeled with 4′,6-diamidino-2-phenylindole (blue). (B) Schematic diagram of RNA-seq analysis of circular RNAs. (C) Venn diagram of circRNA distribution in mock- (ST) and PDCoV-infected (PS) ST cells. (D) Venn diagram of circRNA distribution in pig known genes. (E) The length distribution for circRNAs (length <10,000 nt). (F) Chromosome distribution of the sequenced circRNAs. (G) Clustered heatmap of differentially expressed circRNAs in mock- (ST) and PDCoV-infected (PS) ST cells. (H) Volcano plot constructed using fold change values and *P* values. Red dots represent upregulated circRNAs, and green dots represent downregulated circRNAs.

Using high-throughput RNA-seq, circRNAs from 3 paired PDCoV-infected or mock-infected ST cells were sequenced (Fig. 1A and B). Reads were mapped to the pig reference genome (Sscrofa10.2) using TopHat v2.0.9 (http://ccb.jhu.edu/software/tophat/index.shtml). Unmapped reads were then used to identify circRNAs using find_circ (with CIRI as a supplement) (1, 34). A total of 57,704 distinct circRNAs (each with at least two unique back-spliced reads) were identified in mock- and PDCoV-infected ST cells (Fig. 1C), indicating that circRNAs were abundant in ST cells.

We next annotated the circRNA candidates using the RefSeq database (35). Of 57,704 candidates, we identified 43,050 circRNAs within pig known genes. CircRNAs that were reported mainly consisted of exons (9, 11, 36); however, the annotated circRNAs here were mainly sense-overlapping (24,073, 55.92%), which was consistent with the circRNA features in porcine longissimus muscle reported previously (37); other annotated candidates were located in exons (2,532, 5.88%) or introns (16,445, 38.20%) (Fig. 1D). Among the identified circRNAs, 47.56% were less than 2,500 nucleotides (nt) in length, and the median length was ∼550 nt (Fig. 1E). As shown in Fig. 1F, 57,704 circRNAs were widely and unevenly transcribed from Sus scrofa chromosomes (SSCs). Interestingly, SSC-Y transcribed only 34 circRNAs, 26 of which are encoded by genes *DDX3Y* and *USP9Y* (data not shown). Compared with mock-infected cells, expression analysis identified a number of differentially expressed circRNAs in PDCoV-infected cells (Fig. 1G). In total, 79 differentially expressed circRNAs were identified (Fig. 1H). Of these, 71 circRNAs were upregulated and 8 were downregulated (Fig. 1H), much fewer than previously reported for HSV-1-infected KMB17 cells (16). These results indicate that endogenous circRNAs are likely involved in PDCoV infection.

### Validation of circRNAs differentially expressed in deltacoronavirus-infected cells.

**FIG 2:**
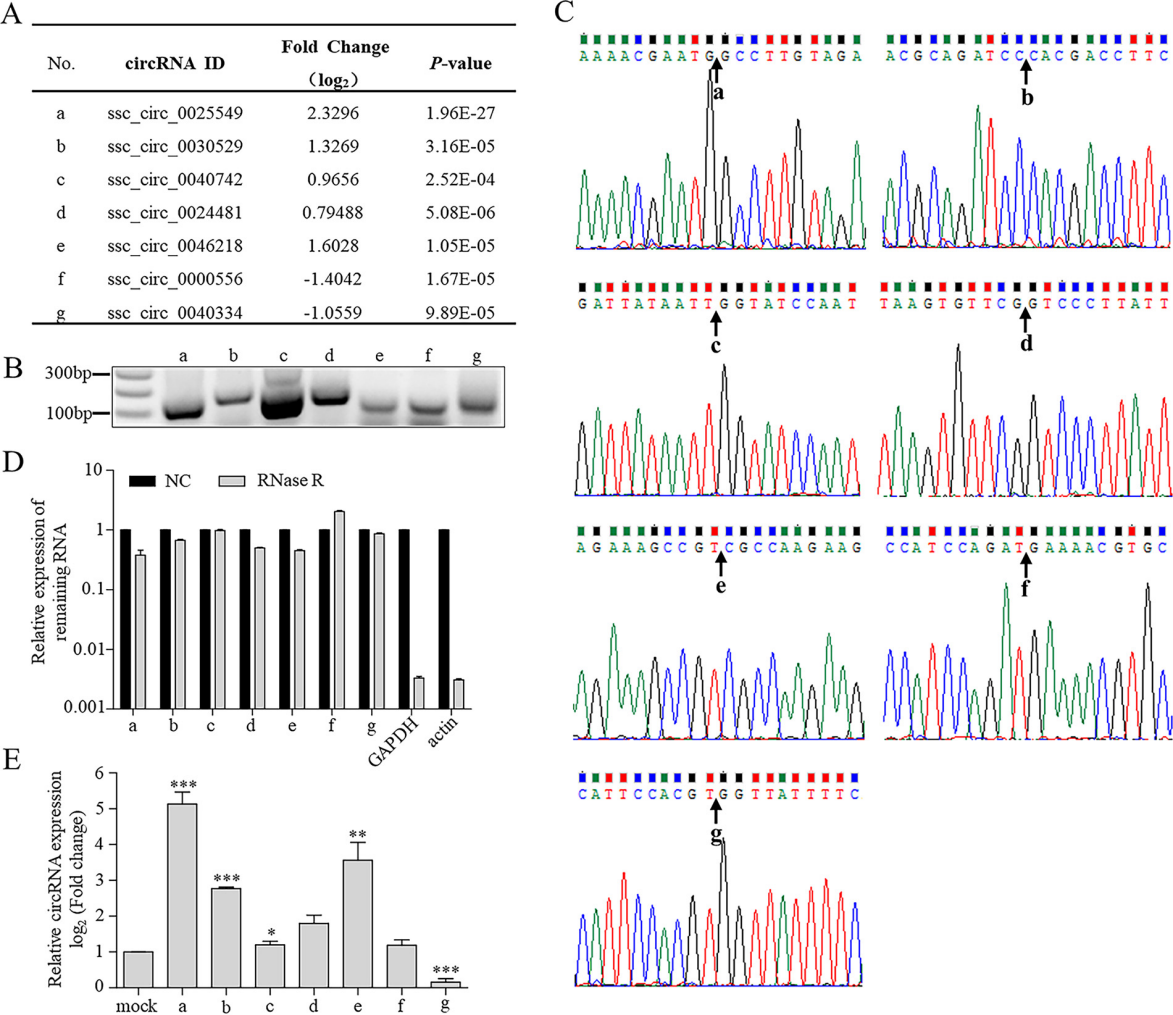
Validation of differentially expressed circRNAs in deltacoronavirus-infected cells. (A) RNA-seq data for seven dysregulated circRNAs, labeled a to g. (B) RT-PCR validation for the seven dysregulated circRNAs. (C) Sanger sequencing validation showing back-splicing events for the seven verified circRNAs. (D) qRT-PCR showing resistance of the seven verified circRNAs to RNase R digestion. Actin and GAPDH mRNAs served as negative controls. Relative expression of circTNFAIP3 was normalized against the mock-treated group. (E) Expression levels of the seven dysregulated circRNAs analyzed by qRT-PCR in ST cells with or without PDCoV infection. ST cells were infected by PDCoV at an MOI of 10 for 11 h, and total RNA was isolated for qRT-PCR analysis. qRT-PCR data were normalized against GAPDH mRNA. Relative expression of circTNFAIP3 was normalized against the mock infection group. Data are represented as the means ± SEM from three independent experiments (*, *P* < 0.05; **, *P* < 0.01; ***, *P* < 0.001; ns, nonsignificant).

To confirm the reliability of RNA-seq results, we designed outward-facing primers to amplify the back-splice junction of these circRNAs. Dysregulated circRNAs with read count of ≥50 and length of ≤1,200 nt were chosen for verification by reverse transcription-PCR (RT-PCR) with Sanger sequencing and quantitative RT-PCR (qRT-PCR) with RNase R treatment. Seven of them (Fig. 2A), including 5 upregulated and 2 downregulated, could be amplified by RT-PCR (Fig. 2B), and their circular structures could be confirmed by sequencing and RNase R treatment (Fig. 2C and D). qRT-PCR analysis revealed that six of the seven identified circRNAs shared the same expression profiles as those in RNA-seq, with ssc_circ_0000556 the exception (Fig. 2E). Similar results were also obtained for mock- and PDCoV-infected IPEC-J2 cells (data not shown).

### Characterization of circTNFAIP3 in tissues.

**FIG 3:**
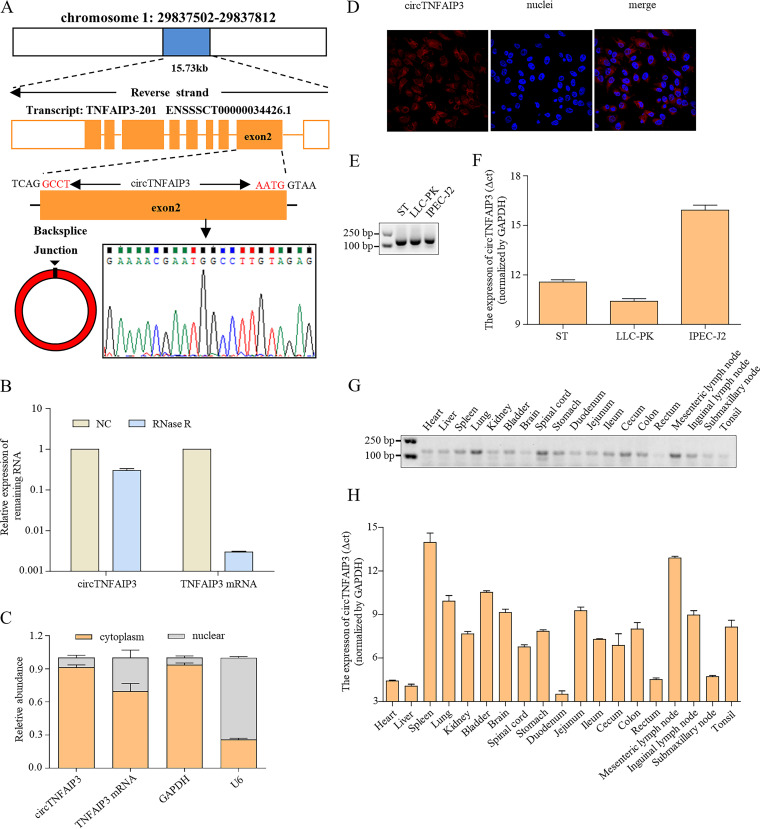
Characterization of circTNFAIP3 expression in tissues. (A) The genomic locus of circTNFAIP3 in the *TNFAIP3* gene within chromosome 1. (B) qRT-PCR analysis of the abundance of circTNFAIP3 and *TNFAIP3* mRNA treated with RNase R in ST cells. The abundances of circTNFAIP3 and *TNFAIP3* mRNA were normalized against the mock treatment group. (C) qRT-PCR analysis of the abundance of circTNFAIP3 and *TNFAIP3* mRNA in the cytoplasm and nucleus of ST cells. GAPDH and U6 mRNAs served as cytoplasmic and nuclear controls, respectively. (D) Fluorescence *in situ* hybridization with junction-specific probes was used to determine the intracellular localization of circTNFAIP3 (red). Nuclei were stained with DAPI (blue). (E) RT-PCR analysis of circTNFAIP3 expression in ST, LLC-PK, and IPEC-J2 cells. (F) qRT-PCR analysis of samples in panel E. (G) RT-PCR analyses of circTNFAIP3 expression in 19 porcine tissues. (H) qRT-PCR analysis of samples in panel G. qRT-PCR data in panels F and H were normalized against GAPDH mRNA. Data are represented as means ± SEM from three independent experiments.

Given that circTNFAIP3 is the most significantly dysregulated among the seven validated circRNAs (Fig. 2A and E), and its homologous protein TNFAIP3, encoded by the linear transcript of *TNFAIP3* gene, plays an important role in inflammation, immunity, and virus infection (38–40), we therefore wondered if the circular transcript of *TNFAIP3* would also be involved in virus infection and selected it for further exploration. Mapping analysis showed that circTNFAIP3 (ssc_circ_0025549, chr1, 29837503 to 29837812) derived from the *TNFAIP3* gene is located on chromosome 1 in pigs, according to the ensemble database (http://ensembl.org) (Fig. 3A). Subsequent structure analysis revealed that circTNFAIP3 is located on the second exon of the *TNFAIP3* gene, flanked by long introns on each side (Fig. 3A). To investigate whether circTNFAIP3 is stable in cells, an RNase R digestion assay was performed. The result showed that circTNFAIP3 was more resistant to digestion than linear transcripts after treatment with exonuclease RNase R, confirming that this RNA species is indeed circular (Fig. 3B). The qRT-PCR analysis and fluorescence *in situ* hybridization (FISH) against circTNFAIP3 showed that the circular form of *TNFAIP3* predominately localized in the cytoplasm (Fig. 3C and D). We also investigated the expression of circTNFAIP3 in different porcine cell lines and tissues. We found that circTNFAIP3 can be detected in three different porcine cell lines (Fig. 3E), and the expression level was higher in LLC-PK cells than in ST cells and IPEC-J2 cells (Fig. 3F). Moreover, circTNFAIP3 can also be detected in 19 porcine tissues, including nine organs, six intestinal tissues, and four lymphoid tissues (Fig. 3G), and the threshold cycle (Δ*C_T_*) value indicated that the highest expression level of circTNFAIP3 is in duodenum (Fig. 3H). These results confirmed that circTNFAIP3 is a widely expressed circRNA in pig tissues.

### Deltacoronavirus replication stimulates circTNFAIP3 expression.

**FIG 4:**
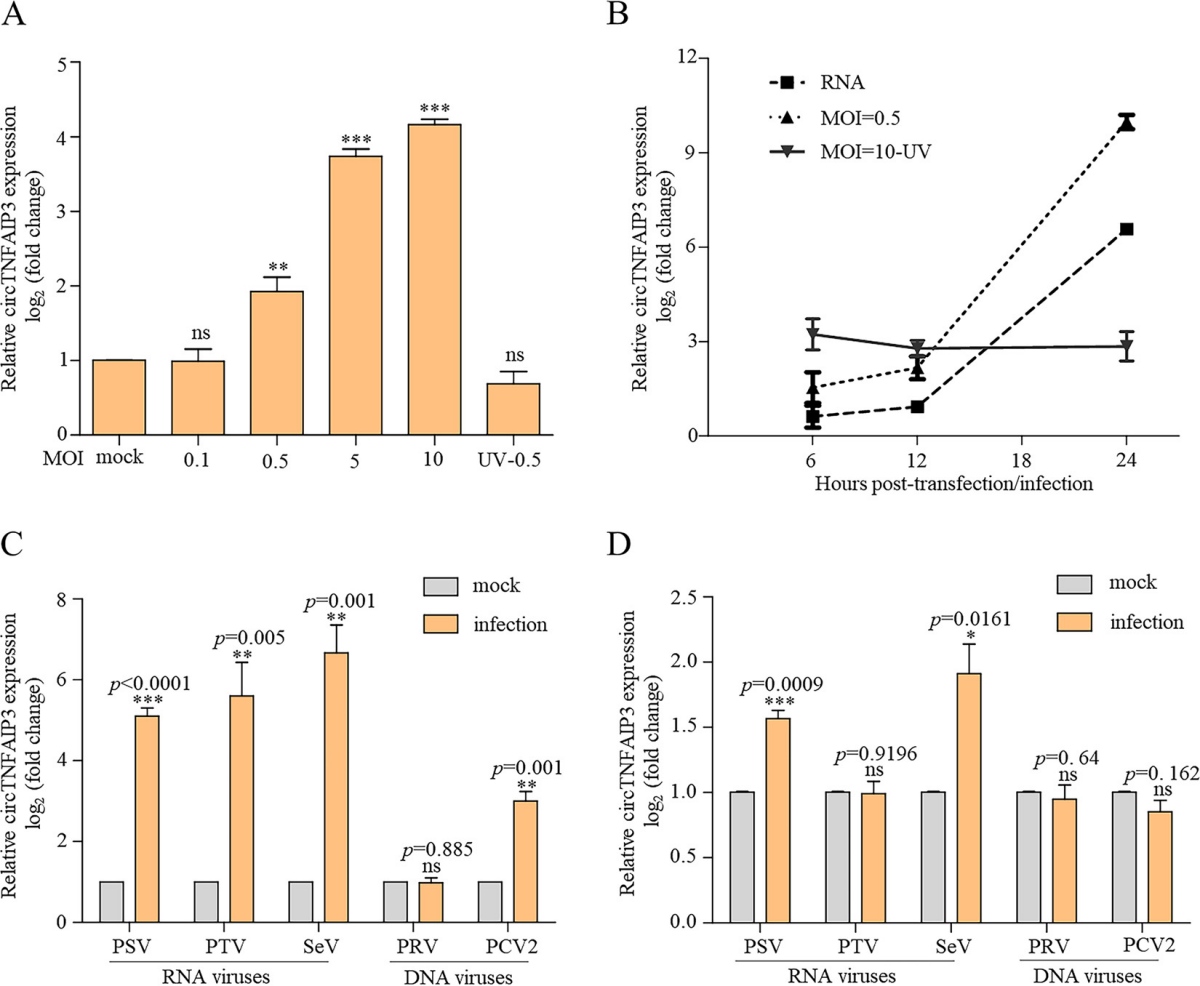
Deltacoronavirus replication stimulates the expression of circTNFAIP3. (A) qRT-PCR analysis of the abundance of circTNFAIP3 in cells with different PDCoV infection doses. ST cells were infected with or without PDCoV at an MOI of 0.1, 0.5, 5, and 10 or with UV-killed PDCoV for 12 h. Total RNA was then isolated for qRT-PCR analysis. (B) Expression curve of circTNFAIP3 in PDCoV-infected and PDCoV genomic RNA-transfected cells. ST cells were transfected with the PDCoV infectious RNA genome or infected with PDCoV at an MOI of 0.5 or 10 (UV killed) for 6, 12, or 24 h. Total RNA was then isolated for qRT-PCR analysis. (C and D) qRT-PCR analysis of the abundance of circTNFAIP3 in ST cells (C) and PK15 cells (D) infected with PSV, PTV, SeV, PRV, or PCV2 at an MOI of 0.5 for 12 h. Relative expression of circTNFAIP3 in panels A to D was normalized against the mock-infection or mock-transfection group. Data are represented as means ± SEM from three independent experiments (*, *P* < 0.05; **, *P* < 0.01; ***, *P* < 0.001; ns, nonsignificant).

To investigate how virus infection affects the expression of endogenous circTNFAIP3, we inoculated ST cells with different doses of PDCoV. The qRT-PCR assay results showed that expression of circTNFAIP3 in PDCoV-infected cells increased gradually with increasing virus dose or replication process, while UV-inactivated PDCoV did not induce an obvious increase in circTNFAIP3 expression (Fig. 4A and B), indicating that circTNFAIP3 expression is dose dependent during PDCoV infection. Interestingly, expression of circTNFAIP3 was increased in cells transfected with the infectious RNA genome of PDCoV (Fig. 4B) but not in cells transfected with pCMV-myc-N, pCMV-myc-E, or pCMV-myc-M (data not shown), suggesting that PDCoV genomic RNA alone could stimulate expression of circTNFAIP3. Moreover, we determined the expression of circTNFAIP3 in cells infected with other RNA viruses, namely, Sendai virus (SeV), porcine sapelovirus (PSV), and porcine teschovirus (PTV), and DNA viruses, porcine pseudorabies virus (PRV) and porcine circovirus type 2 (PCV2). The results showed that RNA virus infection stimulates circTNFAIP3 expression more easily than does DNA virus infection (Fig. 4C and D), demonstrating that RNA viruses tend to upregulate circTNFAIP3 expression in infected cells.

### Deltacoronavirus infection synchronously activates endogenous circTNFAIP3 and TNFAIP3 expression.

**FIG 5:**
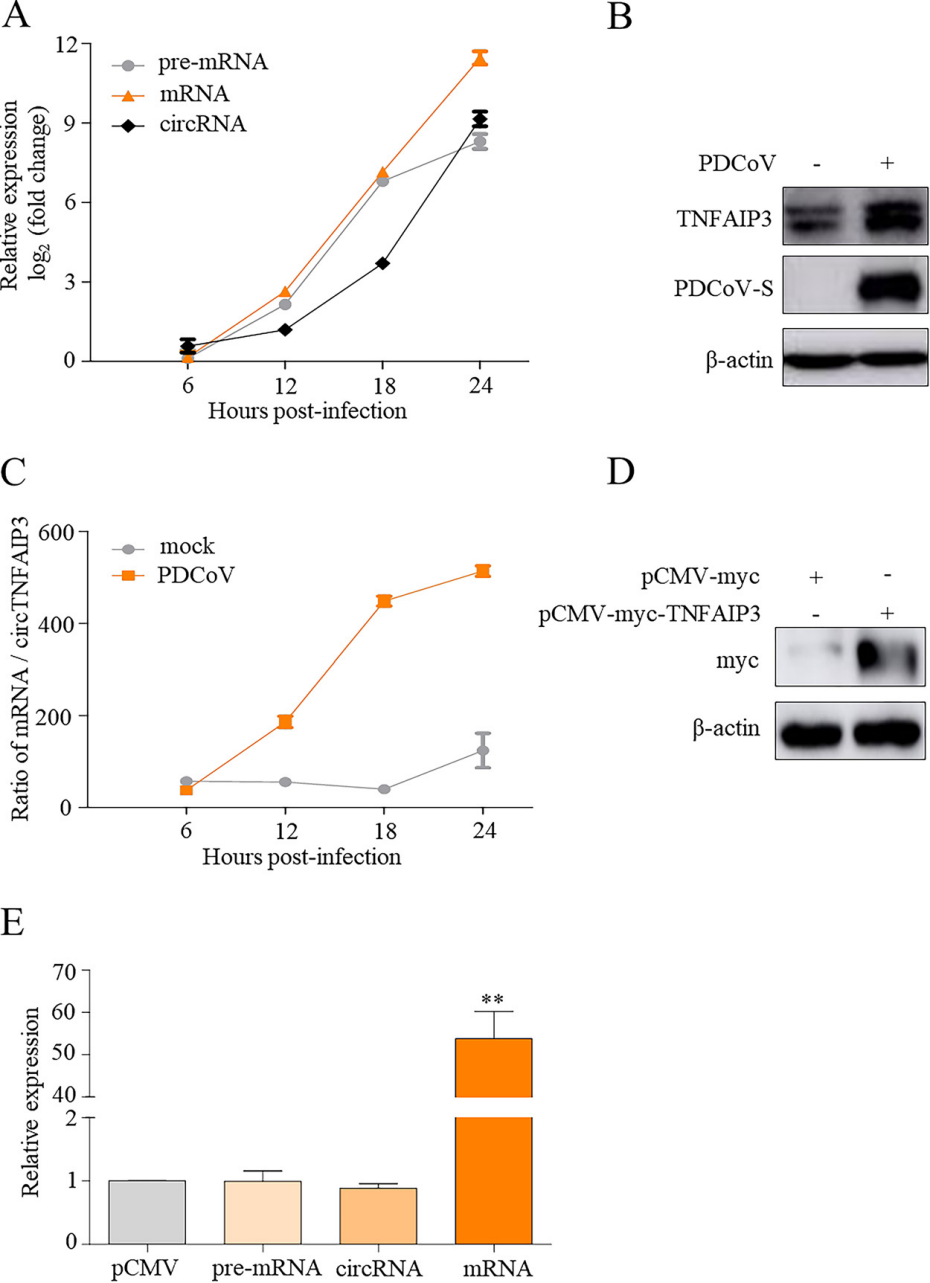
Deltacoronavirus infection synchronously activates endogenous circTNFAIP3 and TNFAIP3 expression. (A) Expression of *TNFAIP3* pre-mRNA, mRNA, and circRNA induced by PDCoV infection. ST cells were infected with or without PDCoV at an MOI of 0.5 for 6, 12, 18, or 24 h. Total RNA was subjected to qRT-PCR to determine the expression of *TNFAIP3* pre-mRNA, mRNA, and circRNA, and expression levels were normalized by the values measured in the mock-infected group. (B) Upregulated expression of the TNFAIP3 protein in PDCoV-infected cells. ST cells in panel A at 12 h postinfection were subjected to Western blotting with an anti-TNFAIP3 antibody recognizing the endogenous TNFAIP3 protein. (C) Ratios of *TNFAIP3* mRNA to circTNFAIP3 in mock- and PDCoV-infected ST cells. Ratios of *TNFAIP3* mRNA to circTNFAIP3 were calculated according to data from panel A. (D) TNFAIP3 expression in pCMV-TNFAIP3-transfected cells. ST cells were transfected with pCMV-TNFAIP3 or pCMV-myc for 24 h and subjected to immunoblotting with anti-myc antibody to detect the expression of TNFAIP3. (E) qRT-PCR analysis of the abundance of pre-mRNA, circRNA, and mRNA of *TNFAIP3* in pCMV-TNFAIP3-transfected cells. Expression levels were normalized by pCMV-transfected group. Data are represented as means ± SEM from three independent experiments (**, *P* < 0.01).

To further explore how deltacoronavirus infection affects the expression of the *TNFAIP3* gene, we investigated the pre-mRNA, circRNA, mRNA, and encoded protein of the *TNFAIP3* gene during virus infection. As shown in Fig. 5A to C, compared with the mock-infected cells, the endogenous pre-mRNA, circRNA, mRNA, and TNFAIP3 protein were synchronously upregulated in PDCoV-infected cells, and the ratio of mRNA to circTNFAIP3 was dramatically increased during the later stages of infection. In TNFAIP3-overexpression cells, TNFAIP3 and TNFAIP3 mRNA were upregulated, whereas *TNFAIP3*-encoded pre-mRNA and circRNA showed no obvious changes without PDCoV infection (Fig. 5D and E). This indicated that TNFAIP3 overexpression did not promote the expression of endogenous circTNFAIP3, while deltacoronavirus infection stimulated the expression of circTNFAIP3 and TNFAIP3 protein.

### Expression of circTNFAIP3 enhances deltacoronavirus replication.

**FIG 6:**
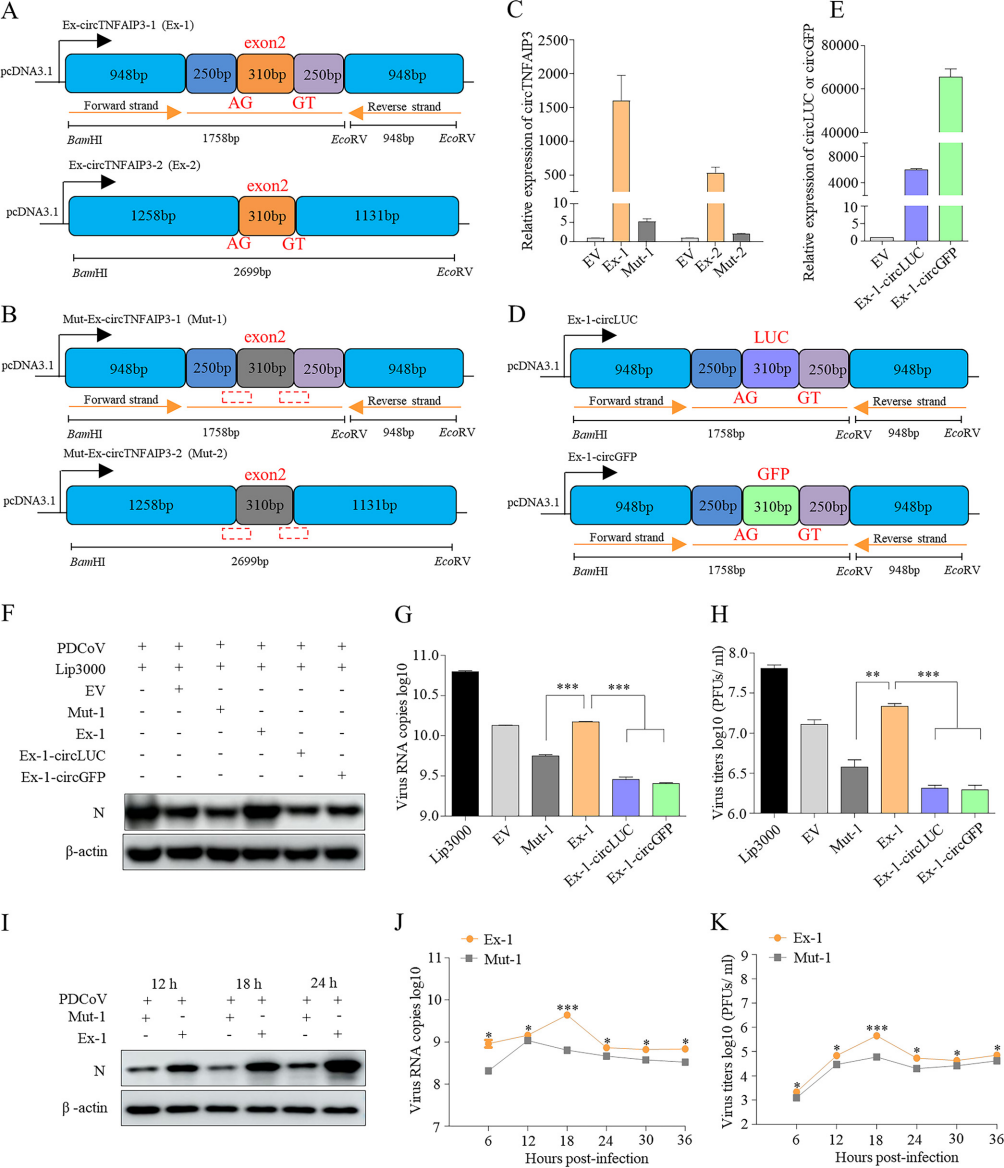
Expression of circTNFAIP3 enhances the replication of deltacoronavirus. (A and B) Schematic representation of the construction of two circTNFAIP3s (A) and the mutant circTNFAIP3 expression plasmids (B). The splice acceptor (AG) and splice donor (GT) were deleted to restrict the expression of circTNFAIP3 in the mutant circTNFAIP3 expression plasmids. (C) qRT-PCR analysis of circTNFAIP3 abundance in ST cells transfected with Ex-circTNFAIP3-1 (Ex-1), Ex-circTNFAIP3-2 (Ex-2), Mut-Ex-circTNFAIP3-1 (Mut-1), Mut-Ex-circTNFAIP3-2 (Mut-2), or pcDNA3.1 (EV), respectively. (D) Schematic representation of the construction of circLUC and circGFP expression plasmids. (E) qRT-PCR analysis of the abundance of circLUC and circGFP in ST cells transfected with Ex-1-circLUC, Ex-1-circGFP, or EV. Expression levels of circRNAs in panels C to E were normalized by EV-transfected group. (F to H) The effect of circTNFAIP3, circLUC, and circGFP on PDCoV replication in ST cells. ST cells were transfected with or without EV, Ex-1, Mut-1, Ex-1-circLUC, or Ex-1-GFP for 24 h, respectively, and infected with PDCoV at an MOI of 0.1 for another 18 h. Cells were collected for WB analysis (F). Supernatant was harvested and subjected to absolute qRT-PCR analysis of PDCoV M gene copies (G). Virus titers were detected by plaque assays (H). (I) Detection of PDCoV N protein in circTNFAIP3-overexpressing ST cells. ST cells were transfected with Ex-1 or Mut-1 for 24 h and infected with PDCoV at an MOI of 0.1 for another 12, 18, and 24 h. Western blotting was performed to examine the expression of PDCoV N protein. (J and K) Replication kinetics of PDCoV in circTNFAIP3-overexpressing cells. ST cells were transfected with Ex-1 or Mut-1 for 24 h and infected with PDCoV for another 6, 12, 18, 24, 30, and 36 h at an MOI of 0.5. Viruses were then harvested and subjected to absolute qRT-PCR analysis of PDCoV M gene copies (J). Virus titers were detected by plaque assays (K). Data are represented as means ± SEM from three independent experiments (*, *P* < 0.05; **, *P* < 0.01; ***, *P* < 0.001; ns, nonsignificant).

To explore the function of circTNFAIP3 during virus infection, we did overexpression experiments first. Two circTNFAIP3 overexpression plasmids, Ex-circTNFAIP3-1 (Ex-1) and Ex-circTNFAIP3-2 (Ex-2), were constructed using two strategies as previously described (9, 10) with some modification (Fig. 6A). By deleting splice acceptor (SA) and splice donor (SD) of Ex-1 and Ex-2, the corresponding mutant plasmids Mut-Ex-circTNFAIP3-1 (Mut-1) and Mut-Ex-circTNFAIP3-2 (Mut-2) losing the circTNFAIP3 overexpression ability were constructed (Fig. 6B). The qRT-PCR showed that Ex-1 and Ex-2 exhibited a 298-fold and 259-fold circTNFAIP3-overexpression enhancement compared with Mut-1 and Mut-2, respectively (Fig. 6C). Considering the higher overexpression efficiency, Ex-1 and Mut-1 were chosen for the following overexpression studies. Meanwhile, two control expression vectors based on Ex-1 framework were constructed by inserting a 310-nt sequence of partial *luciferase* gene or partial *GFP* gene (Fig. 6D), termed Ex-1-circLUC and Ex-1-circGFP, respectively, and they exhibit high expression efficiency in ST cells (Fig. 6E and see Fig. S1A in the supplemental material). Subsequently, ST cells transfected with empty vector pcDNA3.1 (EV), Ex-1, Mut-1, Ex-1-circLUC, Ex-1-circGFP, or Lip3000 (mock transfected) were inoculated with PDCoV as a deltacoronavirus model. Viral proteins, viral RNA copies, and virus titers were measured via Western blot (WB), qRT-PCR, and plaque assays, respectively. As shown in Fig. 6F to K and Fig. S1B, circTNFAIP3 (Ex-1), but not circLUC and circGFP, showed significant promotion of PDCoV N protein level, viral RNA copies, and virus titers compared with Mut-1 overexpression during PDCoV infection; these findings strongly indicated that circTNFAIP3 expression can promote PDCoV replication.

10.1128/mBio.02984-21.1 FIG S1 RT-PCR validation for control circRNAs and cell viability detection. (A) Sanger sequencing validation showing back-splicing events for circLUC and circGFP. (B) Cell viability detection of mock-transfected and plasmid-transfected ST cells using CCK-8 assay. Viability was normalized by the absorbance measured in the mock-transfected control at 450 nm. Data are represented as means ± SEM from three independent experiments (*, *P* < 0.05; **, *P* < 0.01; ***, *P* < 0.001; ns, nonsignificant). Download FIG S1, TIF file, 2.6 MB. Copyright © 2021 Du et al. 2021 Du et al. https://creativecommons.org/licenses/by/4.0/ This content is distributed under the terms of the Creative Commons Attribution 4.0 International license.

**FIG 7:**
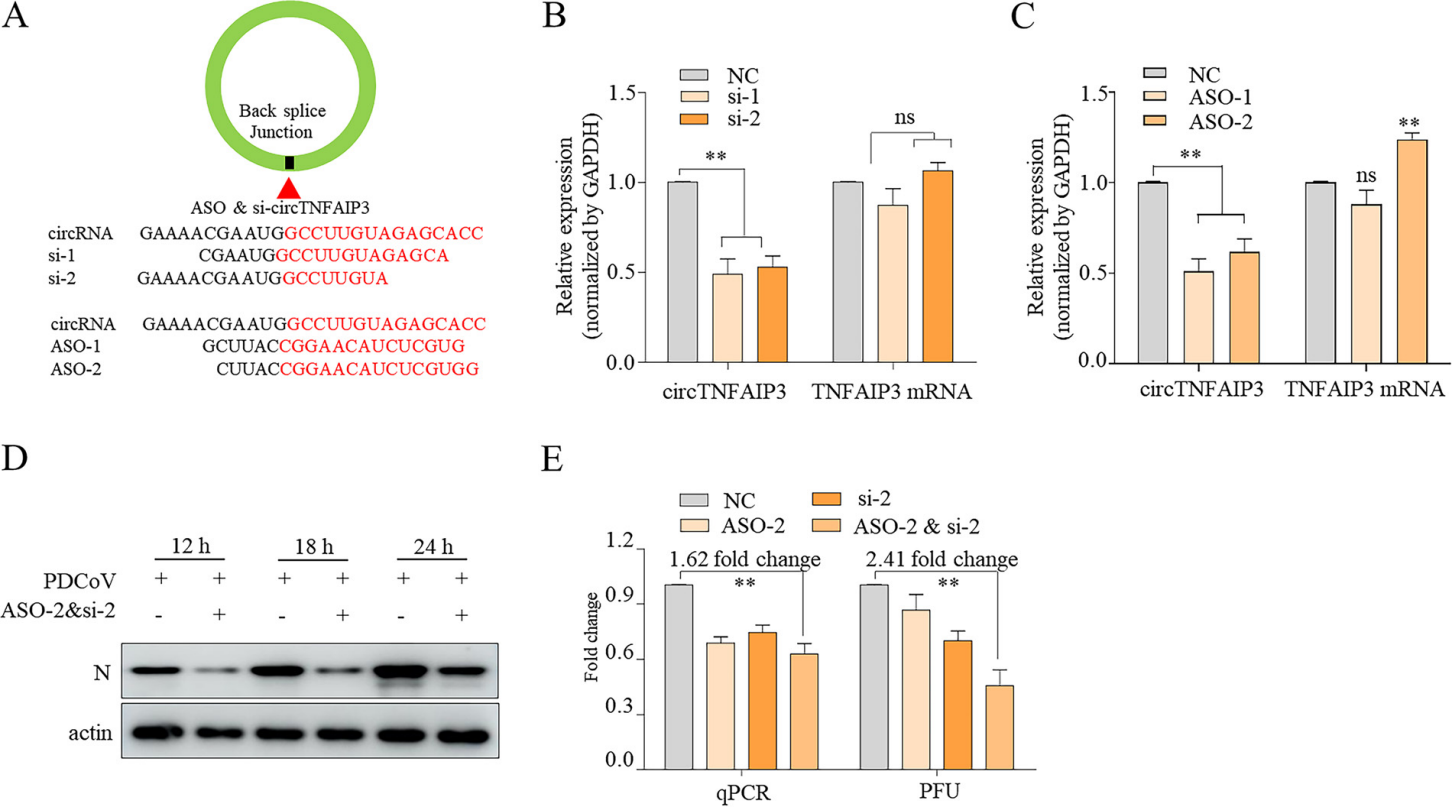
Silencing circTNFAIP3 suppresses the replication of deltacoronavirus. (A) Schematic representation of the target sequences of ASOs and siRNAs specific to the back-splice junction of circTNFAIP3. (B and C) qRT-PCR analysis of the abundance of circTNFAIP3 and *TNFAIP3* mRNA in cells transfected with siRNAs (B), ASOs (C), or control RNAs for 48 h. (D) The expression of PDCoV N protein in circTNFAIP3-silenced ST cells. ST cells were transfected with ASO-2 and si-2 or control RNA for 48 h and were infected with PDCoV at an MOI of 0.1 for another 12, 18, and 24 h. Western blotting was performed to examine the expression of PDCoV N protein. (E) Fold change analysis of PDCoV viral RNA copies and titers in circTNFAIP3-silenced cells. At 48 h posttransfection with ASO-2, si-2, ASO-2 and si-2, or control RNA, ST cells were subsequently infected with PDCoV at an MOI of 0.5 for another 24 h. Cells were then harvested and subjected to absolute qRT-PCR analysis of PDCoV M gene copies, and virus titers were detected by plaque assays. Fold changes were normalized by the values measured in the control RNA-transfected group. Data are represented as means ± SEM from three independent experiments (*, *P* < 0.05; **, *P* < 0.01; ***, *P* < 0.001; ns, nonsignificant).

To further confirm the promotion effect of circTNFAIP3 on PDCoV replication, two small interfering RNAs (siRNAs; si-1 and si-2) targeting cytoplasmic circTNFAIP3 and two antisense oligodeoxynucleotides (ASOs; ASO-1 and ASO-2) targeting nuclear circTNFAIP3 were designed (Fig. 7A) and transfected into ST cells to explore the influence of knocking down endogenous circTNFAIP3 on PDCoV replication. Data showed that siRNAs and ASOs targeting the back-splice junction sequence effectively knocked down the circular transcript but not the linear species of TNFAIP3 (Fig. 7B and C). To knock down cytoplasmic and nuclear circTNFAIP3 simultaneously, si-2 and ASO-2 were chosen to be cotransfected into ST cells for the following knockdown experiments. To further rule out the potential off-target effect of si-2 on the PDCoV genome in cytoplasm, we predicted the target sites of sense and antisense of si-2 on the PDCoV genome using the MiRanda miRNA target prediction tool, and seven potential sites were detected (Fig. S2A). By inserting a 200-nt PDCoV genomic sequence containing these potential target sites into pmirGLO, seven recombinant pmirGLO-PDCoV plasmids were constructed and luciferase assays were performed. Results showed that si-2 did not influence luciferase activities of all seven pmirGLO-PDCoV plasmids, indicating si-2 had no off-target effect on the PDCoV genome (Fig. S2B). Meanwhile, qRT-PCR was used to separately quantify nuclear and cytoplasmic circTNFAIP3 in ST cells transfected with ASO-2 and showed that ASO-2 knocked down only nucleus-located circTNFAIP3 (Fig. S2C and D). Subsequently, ST cells transfected with si-2 and ASO-2 or negative controls were inoculated with PDCoV. PDCoV N protein, viral RNA, and titers were detected to assess the influence of knocking down circTNFAIP3 on PDCoV replication. Results showed that silencing of circTNFAIP3 significantly decreased viral N protein levels, viral RNA copies, and virus titers (Fig. 7D and E). Taken together, we can conclude that circTNFAIP3 is a positive regulator for deltacoronavirus replication.

10.1128/mBio.02984-21.2 FIG S2 si-2 and ASO-2 target circTNFAIP3 specifically. (A) Potential targets of si-2 on PDCoV genome predicted by MiRanda. (B) Luciferase activity of pmirGLO-PDCoV in HEK-293T cells cotransfected with si-2. (C and D) Nuclear-cytoplasmic separation and qRT-PCR analysis of the abundance of GAPDH and U6 (C) and circTNFAIP3 (D) in the cytoplasm and nucleus of ST cells transfected with ASO-2 or control RNA for 48 h. Relative expression of circTNFAIP3 in panel D was normalized by the values measured in the control RNA-transfected group. Data are represented as means ± SEM from three independent experiments (*, *P* < 0.05; **, *P* < 0.01; ***, *P* < 0.001; ns, nonsignificant). Download FIG S2, TIF file, 2.6 MB. Copyright © 2021 Du et al. 2021 Du et al. https://creativecommons.org/licenses/by/4.0/ This content is distributed under the terms of the Creative Commons Attribution 4.0 International license.

### Activity of circTNFAIP3 is independent of miRNA sponging.

**FIG 8:**
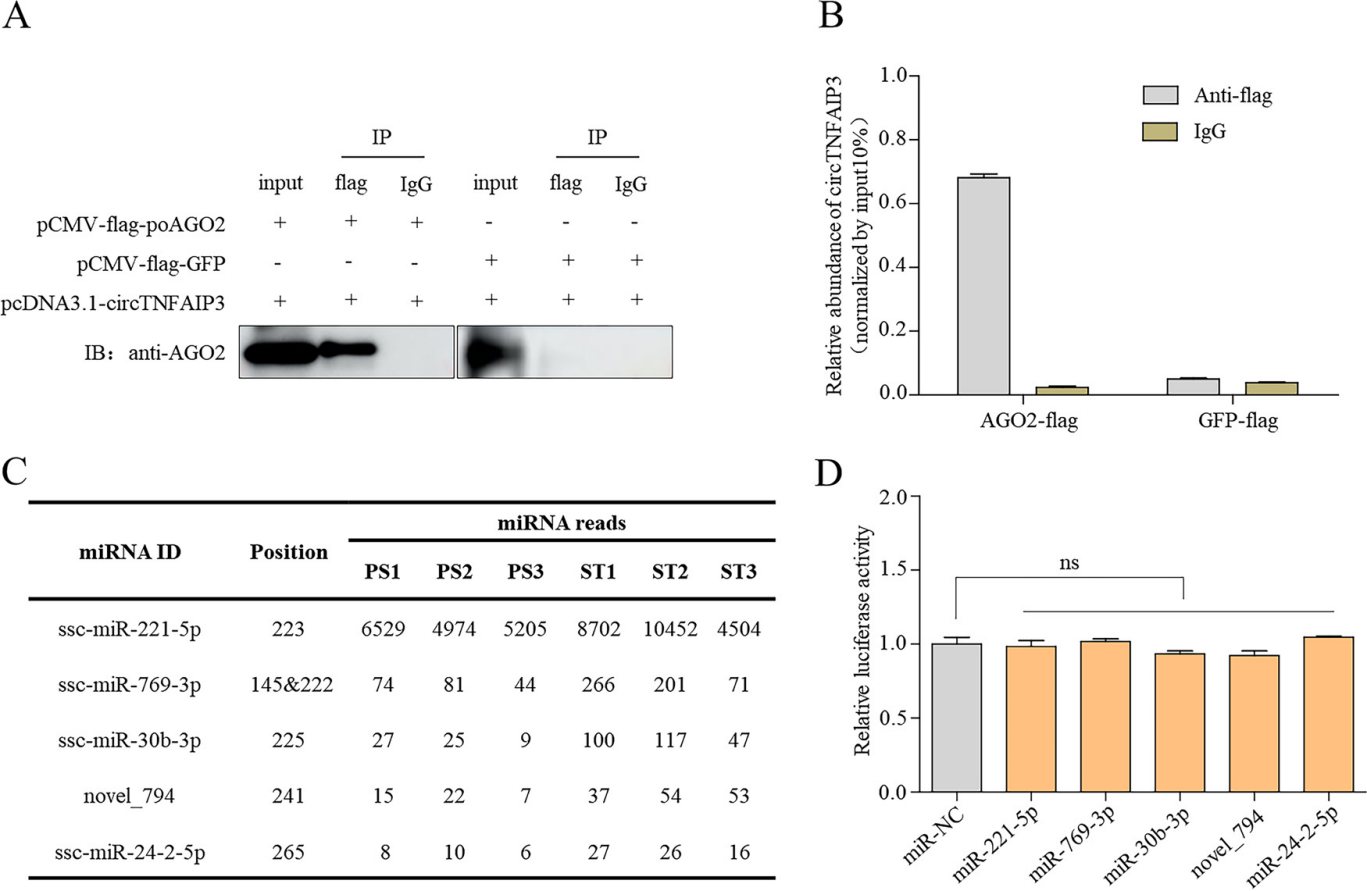
Activity of circTNFAIP3 is independent of miRNA sponging. (A and B) RIP and qRT-PCR experiments were performed in HEK-293T cells. After cotransfection with Ex-1 and AGO2-Flag (or GFP-Flag) for 24 h, HEK-293T cells were lysed for IP and Western blotting (A). IP complex was subjected to qRT-PCR analysis (B). (C) Potential miRNAs binding to circTNFAIP3 were predicted by MiRanda. (D) Luciferase activity of pmirGLO-circTNFAIP3 in HEK-293T cells after cotransfection with miRNA mimics. Data are represented as means ± SEM from three independent experiments (*, *P* < 0.05; **, *P* < 0.01; ***, *P* < 0.001; ns, nonsignificant).

Given that circTNFAIP3 is stable and abundant in the cytoplasm, whether circTNFAIP3 plays a role as an miRNA sponge during PDCoV infection is unknown. To identify the ability of circTNFAIP3 to absorb miRNAs, RNA immunoprecipitation (RIP)-qPCR and luciferase assay were employed. As shown in Fig. 8A and B, circTNFAIP3 were significantly enriched by Flag-porcine AGO2 (poAGO2), but not Flag-GFP (green fluorescent protein), indicating that circTNFAIP3 could bind to homologous AGO2 and has the possibility to be an miRNA sponge. Subsequently, by using the MiRanda miRNA target prediction tool, we predicted the potential miRNAs absorbed by circTNFAIP3 from the miRNA-seq database obtained in this study. A total of 5 miRNAs (Fig. 8C) including novel_794, miR-24-2-5p, miR-30b-3p, miR-221-5p, and miR-769-3p were predicted for further verification by luciferase assay as previously reported (9). Relative luciferase activity showed that, compared with control miRNA, all five miRNAs had no obvious effect on the luciferase activity (Fig. 8D), indicating that even if circTNFAIP3 could bind to AGO2, miRNAs could not be absorbed successfully. Thus, the data imply that circTNFAIP3 modulating PDCoV replication is independent of miRNA sponging.

### CircTNFAIP3 promotes PDCoV replication by inhibiting apoptosis.

**FIG 9:**
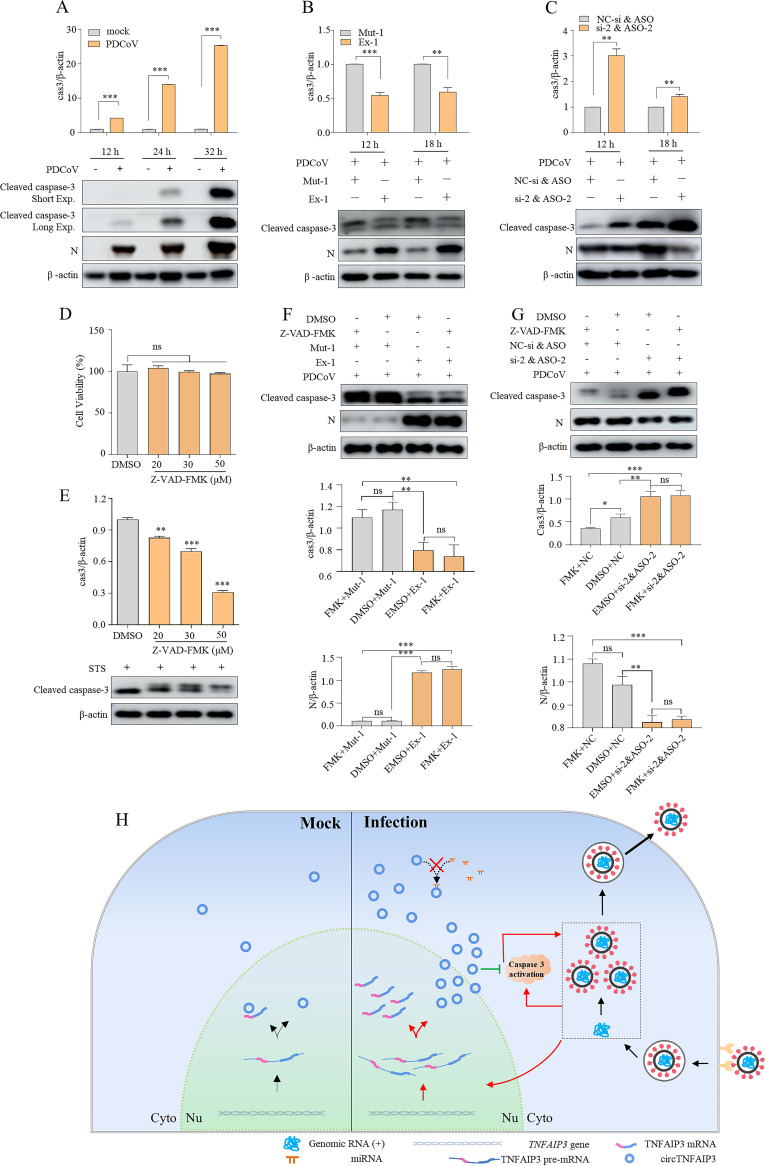
CircTNFAIP3 promotes PDCoV replication by inhibiting apoptosis. (A) PDCoV infection induced apoptosis in ST cells. ST cells were infected with or without PDCoV at an MOI of 0.1 for 12, 24, and 32 h. Cells were harvested at the indicated time points and examined by Western blotting to examine cleaved caspase-3 and PDCoV N protein. (B) Overexpression of circTNFAIP3 suppressed the PDCoV-induced cleavage of caspase-3 and enhanced PDCoV N protein expression. ST cells were transfected with Ex-1 or Mut-1 for 24 h and were infected with PDCoV at an MOI of 0.1 for another 12 or 18 h. Western blotting was performed to examine the expression of PDCoV N protein and cleaved caspase-3. (C) Silencing circTNFAIP3 enhanced the PDCoV-induced cleavage of caspase-3 and suppressed the expression of PDCoV N protein. ST cells were transfected with ASO-2 and si-2 or control RNA for 48 h and were infected with PDCoV at an MOI of 0.1 for another 12 or 18 h. Western blotting was performed to examine the expression of PDCoV N protein and cleaved caspase-3. (D) Detection of ST cell viability. ST cells were treated with different concentrations of Z-VAD-FMK for 24 h, and their viability was measured by CCK-8 assay. (E) Detection of caspase-3 activation in Z-VAD-FMK-treated ST cells. ST cells were pretreated with different concentrations of Z-VAD-FMK for 1 h and then incubated with 0.5 μM STS for another 6 h. Western blotting was performed to examine the expression of cleaved caspase-3. (F) Apoptosis inhibition induced by circTNFAIP3 overexpression in the presence of Z-VAD-FMK. ST cells were transfected with Ex-1 or Mut-1 for 24 h, followed by treatment with Z-VAD-FMK (50 μM) for 1 h, and infected with PDCoV at an MOI of 0.1 for another 8 h. Western blotting was performed to examine the expression of PDCoV N protein and cleaved caspase-3. (G) Apoptosis activated by circTNFAIP3 knockdown in the presence of Z-VAD-FMK. ST cells were transfected with ASO-2 and si-2 or control RNA for 48 h, followed by treatment with Z-VAD-FMK (50 μM) for 1 h, and then infected with PDCoV at an MOI of 0.1 for another 8 h. Western blotting was performed to examine the expression of PDCoV N protein and cleaved caspase-3. (H) Proposed model of circTNFAIP3 based on knowledge of coronavirus life cycle. Data are represented as means ± SEM from three independent experiments (*, *P* < 0.05; **, *P* < 0.01; ***, *P* < 0.001; ns, nonsignificant).

CircRNAs have been reported to regulate cell apoptosis (41–43), and apoptosis is well studied as an antiviral mechanism. Various viruses develop different strategies to limit or utilize apoptosis for benefitting their replication and persistent infection (44, 45), PDCoV has been reported to induce caspase-dependent apoptosis during replication (46). Thus, we wanted to explore if circTNFAIP3 promotes PDCoV replication by regulating apoptosis. As shown in Fig. 9, the apoptosis effector caspase-3 was obviously activated during PDCoV replication (Fig. 9A), and the cleaved caspase-3 is significantly decreased in circTNFAIP3-overexpressing cells but increased in circTNFAIP3-knockdown cells compared with the control (Fig. 9B and C), indicating that circTNFAIP3 inhibits apoptosis induced by PDCoV infection. At the same time, we analyzed the role of circTNFAIP3 in regulating apoptosis in the presence of the apoptosis inhibitor Z-VAD-FMK during PDCoV infection. Data showed that Z-VAD-FMK at a concentration of 20, 30, and 50 μM did not affect the viability of ST cells while it could inhibit staurosporine (STS)-induced apoptosis in a dose-dependent manner (Fig. 9D and E). However, Z-VAD-FMK could not obviously inhibit caspase-3 activation in Mut-1-transfected or si-2- and ASO-2-cotransfected cells with PDCoV infection and also could not strengthen the inhibition of caspase-3 activation in Ex-1-transfected or NC-si-2- and ASO-2-cotransfected cells with PDCoV infection, indicating that the role of Z-VAD-FMK is not obvious in circTNFAIP3-induced apoptosis inhibition during PDCoV infection (Fig. 9F and G). Summarily, circTNFAIP3 contributes to PDCoV replication via acting as an apoptosis inhibitor, not an miRNA sponge (Fig. 9H).

## DISCUSSION

CircRNAs are increasingly being studied in different tissues and cells, especially in human cancer tissues (9, 13, 47–49). Although thousands of circRNAs have been detected in pig tissues (50, 51), their molecular characteristics and expression patterns remain unclear. Previous studies demonstrated that circRNAs have multiple functions as miRNA sponges, in gene transcription, as expression regulators, and in protein coding (36, 52, 53). However, little is known about the role of circRNAs in virus infection. In the present study, we examined circRNA expression profiles during deltacoronavirus infection by RNA-seq, and seven differentially expressed circRNAs were identified in deltacoronavirus-infected cells. Our results provide information on circRNAs that will assist the exploration of coronavirus replication and pathogenesis in the future. To our knowledge, this is the first report on mechanisms of circRNAs regulating deltacoronavirus replication.

We identified 57,704 circRNA candidates in mock- and PDCoV-infected ST cells; the number was more than that of circRNAs in porcine liver tissues (6,366), adipose tissues (13,746), IPEC-J2 cells (26,670), and ovary tissues (38,722) (18, 54–56), but less than that in Jinhua and Landrace pig liver tissues (84,864) (57). It indicates that the circRNA expression in pigs exhibits a complex tissue- or cell-specific characteristic, which is consistent with the circRNA expression pattern in other species (1, 58, 59).

CircRNAs were reported to be involved in cell apoptosis. CircFoxo3 is a bifunctional regulator of apoptosis and can suppress apoptosis through the circFOXO3/miR-29a-3p/SLC25A15 axis (41) or promote cell apoptosis by decreasing interaction between Foxo3 and MDM2 (43). A few studies also uncovered that circGATAD2A promotes H1N1 influenza virus replication by inhibiting autophagy (60), and circPSD3 displays a very pronounced effect on viral RNA abundances in both hepatitis C virus- and dengue virus-infected cells (61). In the present study, a previously undiscovered circRNA, circTNFAIP3, is found to be derived from exon 2 of the porcine *TNFAIP3* gene, expressed widely in piglet tissues (Fig. 3), which is positively correlated with PDCoV replication. Moreover, RNA viruses, rather than DNA viruses, tend to induce a more significant upregulation of circTNFAIP3. Importantly, we proved that circTNFAIP3 favors deltacoronavirus replication by inhibiting apoptosis but not by acting as an miRNA sponge, the most widely reported function of circRNAs (9, 12).

Previously reported circRNAs with multiple functions are predominantly found in the cytoplasm (13, 62). In the present study, circTNFAIP3 was observed in the nucleus as well as the cytoplasm (Fig. 3C and D). Both siRNAs or ASOs could silence the expression of circTNFAIP3, but a combination of siRNAs and ASOs performed better for inhibiting PDCoV replication. It means that circTNFAIP3 localized in the cytoplasm or the nucleus of ST cells plays an important role during PDCoV replication.

An increasing number of ncRNAs have been found to potentially encode proteins (63–65). Circ-ZNF609, associated with heavy polysomes, is translated into a protein that regulates myoblast proliferation (66). Circ-FBXW7, encoding a novel protein of 185 amino acids, suppresses the growth of glioblastoma *in vitro* and *in vivo* (36). In the present study, we also analyzed the protein-encoding ability of circTNFAIP3 and found a potential spanning junction open reading frame with a length of 318 nt, encoding a 105-amino-acid polypeptide. Regrettably, this protein could not be expressed in cells, indicating that circTNFAIP3 is a circRNA with no protein-coding ability.

TNFAIP3 acts as a negative feedback regulator of inflammation and immunity, and this cytoplasmic zinc finger protein was first identified in 1990 as a gene rapidly induced by tumor necrosis factor (TNF) stimulation in human umbilical vein endothelial cells (67). Previous studies showed that TNFAIP3 plays an important role in TNF-induced apoptosis and nuclear factor kappa B (NF-κB)-mediated inflammation (68–71). Moreover, virus infection-induced TNFAIP3 can block the phosphorylation and dimerization of IRF3 and inhibit the TLR3-induced activation of NF-κB and IFN-β (72, 73). In the present study, we found that replication of PDCoV simultaneously enhanced the expression of endogenous circTNFAIP3 and the TNFAIP3 protein, showing that PDCoV hijacks the expression of the *TNFAIP3* gene during infection (Fig. 9H).

In summary, we performed circRNA expression profiling in mock-infected and PDCoV-infected cells and characterized the novel differentially expressed circTNFAIP3 derived from the *TNFAIP3* gene. We confirmed that circTNFAIP3 is strongly associated with various viral infections, especially by RNA viruses, and promotes deltacoronavirus replication via inhibiting apoptosis. Our findings first illustrate that circRNA can act as a negative regulator of apoptosis during RNA virus infection.

## MATERIALS AND METHODS

### Viruses and cells.

PDCoV strain CH-HA3-2017, PSV strain JXXY-a2, PRV strain DX, PCV2 strain HZ0201, PTV, and SeV were stored in our lab (74–77). ST cells (ATCC CRL-1746) were cultured at 37°C in 5% CO_2_ in Dulbecco’s modified Eagle’s medium (DMEM; HyClone, USA) supplemented with 8% heat-inactivated fetal bovine serum (FBS; Gibco, USA). LLC-PK cells (ATCC CL-101) were cultured at 37°C in 5% CO_2_ in minimum essential medium (MEM; HyClone, USA) supplemented with 8% heat-inactivated FBS (Gibco, USA), 1% penicillin-streptomycin (Gibco, USA), 1% HEPES buffer solution (Gibco, USA), and 1% MEM nonessential amino acids (NEAA; Gibco, USA). IPEC-J2 cells, a generous gift from Yaowei Huang at Zhejiang University, China, were cultured at 37°C in 5% CO_2_ supplemented with 8% heat-inactivated FBS (Biological Industries [BI], USA). PK-15 cells (ATCC CCL-33) were cultured in RPMI 1640 medium (Gibco, USA) supplemented with 5% heat-inactivated FBS (Gibco, USA).

### Virus infection and RNA extraction.

For RNA-seq, by optimizing the most appropriate infection dose and collection time point, ST cells fully infected with PDCoV at a multiplicity of infection (MOI) of 10 for 11 h were prepared. For PDCoV infection, DMEM containing 0.2 μg/ml tosylsulfonyl phenylalanyl chloromethyl ketone (TPCK)‐trypsin (Sigma, USA) was employed. Mock-infected cells were placed in the same volume of DMEM, with the same concentration of TPCK-treated trypsin. Total RNA was isolated from each group using SuPerfecTRI total RNA isolation reagent (Pufei, China) according to the manufacturer’s instructions. RNA quality was checked by 1% agarose gel electrophoresis. The purity and concentration of RNA were measured using a NanoPhotometer spectrophotometer (IMPLEN, Germany) and a Qubit RNA assay kit with a Qubit 2.0 fluorimeter (Life Technologies, USA). RNA integrity was assessed using the RNA Nano6000 assay kit of the Bioanalyzer 2100 system (Agilent Technologies, USA). The RNA-seq was performed in PDCoV-infected or mock-infected ST cells, with 3 independent biological experiments. For other infection assays, ST cells were infected with PDCoV at the indicated MOI and harvested at the indicated time. Heart, liver, spleen, lung, kidney, bladder, brain, spinal cord, stomach, duodenum, jejunum, ileum, cecum, colon, rectum, mesenteric lymph node, inguinal lymph node, submaxillary node, and tonsil were collected from 3-month-old healthy pigs. Total RNA from these samples for RT-PCR and qRT-PCR was isolated using RNA Isolater total RNA extraction reagent (Vazyme, China). Nuclear and cytoplasmic fractions for qRT-PCR were isolated using NE-PER nuclear and cytoplasmic extraction reagents (Thermo Scientific, USA).

### CircRNA-seq analysis.

Total RNA from each sample was treated with an Epicentre Ribo-Zero rRNA removal kit (Epicentre Biotechnologies, USA) and RNase R exonuclease (Epicentre Biotechnologies, USA) to obtain rRNA-depleted and RNase R-digested RNAs. Subsequently, sequencing libraries were generated with an NEBNext Ultra Directional RNA library prep kit for Illumina (New England Biolabs [NEB], USA) following the manufacturer’s recommendations. Briefly, fragmentation was carried out using divalent cations under an elevated temperature in NEBNext first-strand synthesis reaction buffer. First-strand cDNA was synthesized using random hexamer primer and Moloney murine leukemia virus (M-MuLV) reverse transcriptase (RNase H^−^). Second-strand cDNA synthesis was then performed using DNA polymerase I and RNase H. In the reaction buffer, deoxynucleoside triphosphates (dNTPs) with dTTP were replaced by dUTP. Remaining overhangs were converted into blunt ends via exonuclease/polymerase activities. After adenylation of the 3′ ends of DNA fragments, NEBNext Adaptor with a hairpin loop structure was ligated in preparation for hybridization. To preferentially select cDNA fragments of 150 to 200 bp in length, fragments were purified with an AMPure XP system (Beckman Coulter, Beverly, MA, USA). Next, 3 μl of USER enzyme (NEB, USA) was incubated with size-selected, adaptor-ligated cDNAs at 37°C for 15 min, followed by 5 min at 95°C, prior to PCR. After PCR amplification, the library was purified (using an AMPure XP system) and then qualified by an Agilent Bioanalyzer 2100 system. Clustering was performed on a cBot cluster generation system using a HiSeq PE cluster kit v4 cBot (Illumina, USA) according to the manufacturer’s instructions. After cluster generation, library preparations were sequenced on a HiSeq X 10 PE150 platform, and 150-bp paired-end reads were generated.

### Identification and quantification of circular RNAs.

Raw data (raw reads in FASTA format) were first processed by a custom perl script, and clean data (clean reads) were obtained after removing adaptor-containing reads, poly(N)-containing reads, and low-quality reads. The reference genome and gene annotation information were downloaded from the genome website (http://www.ensembl.org). The indexed reference genome was built using Bowtie v2.0.6, and paired-end clean reads were aligned to the reference genome using TopHat v2.0.9 (http://ccb.jhu.edu/software/tophat/index.shtml). Unmapped reads were kept, and 20-mers from the 5′ and 3′ ends of these reads were extracted and aligned independently against reference sequences by Bowtie v2.0.6. Anchor sequences were extended by find_circ (1) such that the complete read aligned and breakpoints were flanked by GU/AG splice sites. Back-spliced reads with at least two supporting reads were then annotated as circRNAs. We also used CIRI (34) to identify and characterize circRNAs. The final circRNA library was based on the combined results of find_circ and CIRI. Expression levels of circRNAs were normalized using the transcripts per million (TPM) method using the following criteria: normalized expression = (mapped reads)/(total reads) × 1,000,000. Differential expression between groups was determined using DESeq2 (version 1.6.3) (78), and *P* values were adjusted by the Benjamini and Hochberg method. By default, the threshold for corrected *P* values for differential expression was set to 0.05.

### Library construction, sequencing, and data analysis of miRNA.

Library construction, sequencing, and data analysis were entrusted to Novogene (Tianjin, China). Sequencing libraries were generated using NEBNext Multiplex according to the manufacturer’s recommendations. The small RNA (sRNA) molecules were ligated to a 5′ adaptor and a 3′ adaptor using T4 RNA ligase 1. Then, first-strand cDNA was synthesized using M-MuLV reverse transcriptase. PCR amplification was performed using LongAmp *Taq* 2× master mix (NEB, USA). At last, library quality was assessed on the Agilent Bioanalyzer 2100 system using DNA High-Sensitivity Chips (Agilent Technologies, USA). After cluster generation, the library preparations were sequenced on an Illumina HiSeq 2500/2000 platform, and 50-bp single-end reads were generated.

Raw reads were first processed through custom perl and python scripts. In this step, clean reads were obtained by removing low-quality reads. The small RNA tags were mapped to reference sequence by Bowtie without mismatch to analyze their expression and distribution on the reference. Using miRBase (http://www.mirbase.org) as reference, modified software miRDeep2 was used to obtain the potential miRNA, and miREvo and miRDeep2 were integrated to predict novel miRNA (79, 80). Expression analysis of miRNAs was performed using the same method as used for circRNAs.

### RT-PCR and qRT-PCR.

Extracted RNAs were used for RT-PCR or qRT-PCR. For relative quantitative analysis, cDNAs were synthesized using HiScript II Q RT SuperMix for qPCR with genomic DNA (gDNA) eraser (Vazyme, China), and the real-time PCR assays were performed using AceQ qPCR SYBR green master mix (Vazyme, China). The relative fold change was calculated by the 2^−ΔΔ^*^CT^* method, and glyceraldehyde-3-phosphate dehydrogenase (GAPDH) was used to normalize the relative expression levels of circular and linear RNAs. For absolute quantitative analysis, cDNAs were synthesized using HiScript II Q RT SuperMix for qPCR (Vazyme, China), and the real-time PCR assays were performed using AceQ qPCR probe master mix (Vazyme, China). The probe for the PDCoV M gene was 5′-FAM (6-carboxyfluorescein)-CACACCAGTCGTTAAGCATGGCAAGCT-BHQ (black hole quencher)-3′ (81). All RT-PCR and qRT-PCR primers used in this study are listed in Table 1.

**TABLE 1:** Primers used for PCR and qPCR

Primer	Sequence (5′ to 3′)
25549-F	TCATCCACAAAGCTCTCATCGACAG
25549-R	TGCTCAGCCATGGTGCTCTACAAG
30529-F	CTCAGTGCCCGTGCCTTACATCATT
30529-R	TCACCACAACCTCATAGGGGTCAGC
40742-F	GGAGCTGGAAATTGAAAAGGAG
40742-R	GGAGAGTGCCTCTTCTATTGGATAC
24481-F	GGGAGGACTTCACTTGCTTCTGG
24481-R	GACTCCAAAGACTCACGGGAAATAA
46218-F	CTTGGCTGCCTTCGCCTTCTTC
46218-R	AAGCCTAAAGCCGCAAAACCCAA
556-F	AACGTGATCTCCACAAGAAACCCAT
556-R	CCTGGTCATACTGGTCAGTGTAAAA
40334-F	GTACCCGTTGATGGCTTCAAACCTG
40334-R	TCCACGTCATACGGTGGTGACAGAG
TNFAIP3-mRNA-F	TTTGTCCCCCTGGTGACCCTGA
TNFAIP3-mRNA-R	TTTCGGGATCTGTCAAGAAGTGAAC
TNFAIP3-Pre-mRNA-F	GGTAATGACAAGATCAAACACTGGG
TNFAIP3-Pre-mRNA-R	TCAAATACAAAGCCAGGGGAAG
GAPDH-F	TGGTGAAGGTCGGAGTGAAC
GAPDH-R	GGAAGATGGTGATGGGATTTC
β-actin-F	TCATCACCATCGGCAACG
β-actin-R	TTGAAGGTGGTCTCGTGGAT
U6-F	ATTGGAACGATACAGAGAAGATT
U6-R	GGAACGCTTCACGAATTTG
circLUC-F-	ACCTTCGTGACTTCCCATTTGCCA
circLUC-R	TTCAGCAGCTCGCGCTCGTTGTA
circGFP-F	TCAAGGACGACGGCAACTACAAG
circGFP-R	GGTGCAGATGAACTTCAGGGTCA
PDCoV-M-F	ATCGACCACATGGCTCCAA
PDCoV-M-R	CAGCTCTTGCCCATGTAGCTT

### Immunofluorescence assay (IFA).

ST cells in 6-well plates with 80% confluence were mock infected or infected with PDCoV at an MOI of 10 for 11 h. Cells were washed three times with phosphate-buffered saline (PBS) and fixed with formaldehyde and acetone (1:1) at 4°C for 30 min. After blocking in 5% skim milk for 1 h at room temperature, cells were incubated with rabbit polyclonal antibody recognizing the PDCoV S protein at 37°C for 1 h and subsequently incubated with a fluorescein isothiocyanate (FITC)-labeled goat anti-rabbit secondary antibody at 37°C for 1 h, followed by treatment with 4′,6-diamidino-2-phenylindole (DAPI) at room temperature for another 10 min. Fluorescence images were captured using a Nikon TI-S inverted fluorescence microscope (Nikon, Japan).

### FISH.

Cy3-labeled circTNFAIP3 probe was synthesized by RiboBio (Guangzhou, China). RNA FISH was performed using a fluorescent *in situ* hybridization kit (RiboBio, China) following the manufacturer’s instructions.

### RNase R digestion.

Total RNA (2 mg) was incubated for 20 min at 37°C with or without 3 U/mg of RNase R (Epicentre Biotechnologies, USA). The resulting RNA was purified by a second phenol-chloroform extraction.

### PDCoV purification.

Cultured PDCoV was concentrated by ultracentrifugation in a Ty50.2 rotor (Beckman Coulter, CA, USA) through a 15% (wt/vol) sucrose gradient for 2 h at 50,000 × *g*. The pellet was resuspended in 200 μl of NTE buffer (0.05 M Tris-HCl, 0.15 M NaCl, 15 mM CaCl_2_, pH 6.5) overnight. The PDCoV-infectious RNA genome was isolated from the purified virions.

### Plasmid construction.

Two strategies were performed to construct overexpression plasmids for circTNFAIP3 as previously described (9, 10). In the first strategy, we inserted *TNFAIP3* exon 2 along with the upstream flanking sequence (1,198 nt) and downstream flanking sequence (250 nt) into pcDNA3.1 and then copied part of the upstream flanking sequence (948 nt) and inserted it downstream in an inverted orientation. This vector was named Ex-circTNFAIP3-1 (Ex-1). In the second strategy, we inserted a 2,699-nt region of the *TNFAIP3* gene, including a 1,258-nt upstream sequence, the full-length exon 2 (310 nt), and a 1,131- nt downstream sequence, into pcDNA3.1, to generate Ex-circTNFAIP3-2 (Ex-2). The corresponding mutants, Mut-Ex-circTNFAIP3-1 (Mut-1) and Mut-Ex-circTNFAIP3-2 (Mut-2), which lack the ability to overexpress circTNFAIP3, were constructed by deleting the splice acceptor (AG) and splice donor (GT), respectively. Ex-1-circLUC or Ex-1-circGFP was constructed by replacing the 310-nt circTNFAIP3 region of Ex-1 with a 310-nt *luciferase* or *GFP* gene, respectively. The TNFAIP3 overexpression vector was constructed by inserting the full-length coding region (CDS; 2,358 nt) into pCMV-myc. The AGO2-Flag and GFP-Flag expression vectors were constructed by inserting the coding region (2,583 nt and 717 nt) into pCMV-Flag. The pmirGLO-circTNFAIP3 and pmirGLO-PDCoV vectors were constructed by inserting the full-length exon 2 (310 nt) or 7 PDCoV genomes with the length of 200 nt containing the potential target sequences of si-2 into pmirGLO vector, respectively. All primers used to construct plasmids are listed in Table 2. All gene cloning was performed using Phanta Max Super-Fidelity DNA polymerase (Vazyme, China), and all constructs were verified by DNA sequencing.

**TABLE 2:** Primers for plasmid construction

Primer	Sequence (5′ to 3′)
ex-circTNFAIP3-1-up-F	CGCGGATCCGAAATCAGGATGGATGACAGGGCAC
ex-circTNFAIP3-1-up-R	CGGATATCTGGTGTCTAGAAATGCAGTCCCCAA
ex-circTNFAIP3-1-down-F	CGGATATC AGGAGGGGAATAACCCGTGTTTTCA
ex-circTNFAIP3-1-down-R	CCGCTCGAGGAAATCAGGATGGATGACAGGGCAC
ex-circTNFAIP3-2-F	CTTGGTACCGAGCTCGGATCCTGCAGGGAAACTTCCTAGGGCC
ex-circTNFAIP3-2-R	GCCGCCACTGTGCTGGATATCTTTGATTGGTATGGTTTAGGAACGTCAC
Mut-ex-circTNFAIP3-1/2-AG-F	CTTTCCCTCCTCTTCGCCTTGTAGAGCACCA
Mut-ex-circTNFAIP3-1/2-AG-R	TGGTGCTCTACAAGGCGAAGAGGAGGGAAAG
Mut-ex-circTNFAIP3-1/2-GT-F	CACTGAAAACGAATGAAGACTTGCTTTTGTC
Mut-ex-circTNFAIP3-1/2-GT-R	GACAAAAGCAAGTCTTCATTCGTTTTCAGTG
TNFAIP3-F	ATGGCCATGGAGGCCCGAATTCGGATGGCTGAGCAACTCCTTCCCCTG
TNFAIP3-R	GAGATCTCGGTCGACCGAATTCCTAGCCATACATCTGCTTGAATTG
AGO2-F	CCCAAGCTTATGTACTCGGGAGCCGGCCC
AGO2-R	CCGGAATTCTCACGCAAAGTACATGGTGCGCAG
GFP-F	CCCAAGCTTGTGAGCAAGGGCGAGGAGCTGTT
GFP-R	CCGGAATTCCTACTTGTACAGCTCGTCCATGCCGA
pmirGLO-circTNFAIP3-F	CCGCTCGAGGCCTTGTAGAGCACCATGGCTGAGC
pmirGLO-circTNFAIP3-R	CCGCTCGAGCATTCGTTTTCAGTGCCACAAGCTTCC
ex-1-circLUC/GFP-up-F	CGCGGATCCGAAATCAGGATGGATGACAGGGCAC
ex-1-circLUC/GFP-up-R	CCGGAATTCCTGAAGAGGAGGGAAAGAAAACCCC
ex-1-circLUC-F	TTCCCTCCTCTTCAGAGCTAACGACATCTACAACGAGCGC
ex-1-circLUC-R	TGCTGGATATCTGCAGAATTCGCCCTTGGGCAATCCGGTACT
ex-1-circGFP-F	TTCCCTCCTCTTCAGAGCTGTTCACCGGGGTGGTG
ex-1-circGFP-R	TGCTGGATATCTGCAGAATTCCGGGTCTTGTAGTTGCCGTCGTC
ex-1-circLUC-down-F	GGATTGCCCAAGGGCGTAAGACTTGCTTTTGTCAGTGGGGTGG
ex-1-circGFP-down-F	CAACTACAAGACCCGGTAAGACTTGCTTTTGTCAGTGGGGTGG
ex-1-circLUC/GFP-down-R	AACGGGCCCTCTAGACTCGAGGAAATCAGGATGGATGACAGGGCAC
pmirGLO-PDCoV-1-F	CCGCTCGAGACCCTGGTATTTGCATTTCATTTAC
pmirGLO-PDCoV-1-R	ACGCGTCGACAAGAACAGCAACAACGCATAGTATG
pmirGLO-PDCoV-2-F	CCGCTCGAGACTTTGGACTTATGATATTGTCTGATG
pmirGLO-PDCoV-2-R	ACGCGTCGACCACAGTATGCTGTGAGCAAAATTC
pmirGLO-PDCoV-3-F	CCGCTCGAGACTTTTTGTTTTTTTAGCACCATTC
pmirGLO-PDCoV-3-R	ACGCGTCGACGTAGGGAGTCTTGACCGTCAAAA
pmirGLO-PDCoV-4-F	CCGCTCGAGCTGTTCTTGCAGTTCAGCGATTCTT
pmirGLO-PDCoV-4-R	ACGCGTCGACAGCGAAGTAAAACAATGCCACATTC
pmirGLO-PDCoV-5-F	CCGCTCGAGGATCCAATTGAGAACCCATCCTT
pmirGLO-PDCoV-5-R	ACGCGTCGACCCATTACCTGGTTGTGTAATAATAAAA
pmirGLO-PDCoV-6-F	CCGCTCGAGCCAACTTCAAGGGTGACTACAAT
pmirGLO-PDCoV-6-R	ACGCGTCGACTAGAACCATGATGCCATTGTAATAC
pmirGLO-PDCoV-7-F	CCGCTCGAGGACTTTTCAACCCTCAATGCAGA
pmirGLO-PDCoV-7-R	ACGCGTCGACACAAGTGTAGAAGGAGTAACAGCGC

### Transfection of plasmids, ASOs, and siRNAs.

ASOs, siRNAs targeting the back-spliced junction of circTNFAIP3, and their nontargeting negative controls were synthesized by RiboBio (Guangzhou, China) and GenePharma (Shanghai, China), respectively. The ASO and siRNA sequences are presented in Fig. 7A. Transient transfection of plasmids, ASOs, and siRNAs was performed using a Lipofectamine 3000 kit (Invitrogen, USA) according to the manufacturer’s instructions.

### Plaque assay.

ST cells were grown to a 95% confluency monolayer in a 12-well plate and then incubated with PDCoV at 37°C for 1 h. Subsequently, an equal volume of DMEM and low-melting-point Agarose II (Thermo Scientific, USA) were mixed and added to the surface of ST cells. At 48 h postinfection (hpi), cells were fixed with 10% neutral buffered formaldehyde at room temperature for 30 min and then stained by 5% crystal violet solution for 2 h, and plaques were accumulated to calculate the titer of harvested PDCoV.

### Western blotting.

Total proteins were extracted using lysis buffer consisting of 2% sodium dodecyl sulfate (SDS), 1% Triton X-100, 50 mM Tris-HCl, and 150 mM NaCl (pH 7.5), separated by 10% SDS-PAGE, and transferred to a nitrocellulose membrane. The membrane was then blocked with 5% skim milk and incubated with primary antibodies at 4°C overnight. After hybridization with horseradish peroxidase (HRP)-conjugated secondary antibodies, the membrane was visualized using enhanced chemiluminescence (ECL) reagents (Bio-Rad, Hercules, CA, USA), and a Quantity One system (Bio-Rad, USA) was applied for analysis. The primary antibodies were anti-myc (Huabio, China), anti-TNFAIP3 (Proteintech, USA), anti-β-actin (Huabio, China), anti-caspase-3 (Cell Signaling Technology [CST], USA), anti-AGO2 (Abcam, USA), and anti-PDCoV N protein (Medgene Labs, USA), and the anti-PDCoV S protein polyclonal antibody was stored in our lab.

### RNA immunoprecipitation.

HEK-293T cells were transfected with Ex-1 and AGO2-Flag (or GFP-Flag) plasmids for 36 h. Approximately 6 × 10^6^ cells were then pelleted and resuspended with 600 μl NP-40 lysis buffer plus protease and RNase inhibitors. The cell lysates (500 μl) were incubated with 5 mg of control mouse IgG or antibody against Flag peptide (Huabio, China)-coated beads with rotation at 4°C overnight, respectively. Then, the RIP complex was concentrated, and RNA was extracted as mentioned above. The abundance of circTNFAIP3 level was detected by RT-qPCR assay.

### Luciferase reporter assay.

HEK-293T cells were seeded in 24-well plates 24 h before transfection. Cells were cotransfected with a mixture of 300 ng vector (pmirGLO-circTNFAIP3 or pmirGLO-PDCoV) and 1.5 μl RNA (miRNA mimics or si-2) (20 μM) using jetPRIME (Polyplus, France) according to the manufacturer’s instructions. The 5 miRNA mimics were synthesized by GenePharma (Shanghai, China). After 36 h, the luciferase activity was measured with a dual luciferase reporter assay system (Beyotime Biotechnology, China) according to the manufacturer’s instructions. The firefly luciferase activity was normalized with its corresponding *Renilla* luciferase activity.

### CCK-8 assay.

ST cells cultured on 96-well plates were transfected with PGL3, pmirGLO, pCDH, pCAGGS, pCMV, pcDNA3.1, Ex-1, Mut-1, Ex-1-circLUC, or Ex-1-circGFP and mock transfected (Lip3000) for 24 h. Blank wells (medium without cells) were added with the same amount of culture medium. Then, 10 μl/well (96-well plate) CCK-8 solution (Beyotime Biotechnology, China) was added to each well. The treated cells were protected from light and incubated for 3 h at 37°C. Viability was normalized by the absorbance measured in the mock-transfected control at 450 nm. The viability of ST cells treated with dimethyl sulfoxide (DMSO) (Sigma, USA) and Z-VAD-FMK (Beyotime Biotechnology, China) was measured according to the above-mentioned method. Data are represented as means ± standard errors of the means (SEM) from three independent experiments.

### Statistical analysis.

All statistical analyses were performed using GraphPad Prism 5.0 software. Quantitative data are expressed as means ± standard errors of the means (SEM). Statistically significant differences were evaluated using Student’s *t* tests, and *P* < 0.05 was considered statistically significant.

### Data availability.

The RNA-seq data set of circRNA and miRNA used in this paper has been deposited at Gene Expression Omnibus (https://www.ncbi.nlm.nih.gov/geo/) under the accession numbers GSE147188 and GSE176550, respectively.
