# Supplementary figures and images for: A Previously Undiscovered Circular RNA, circTNFAIP3, and Its Role in Coronavirus Replication
Source: mBio. 2021 Nov 16;12(6):e02984-21. doi: 10.1128/mBio.02984-21 (PMC8593679; doi:10.1128/mBio.02984-21)

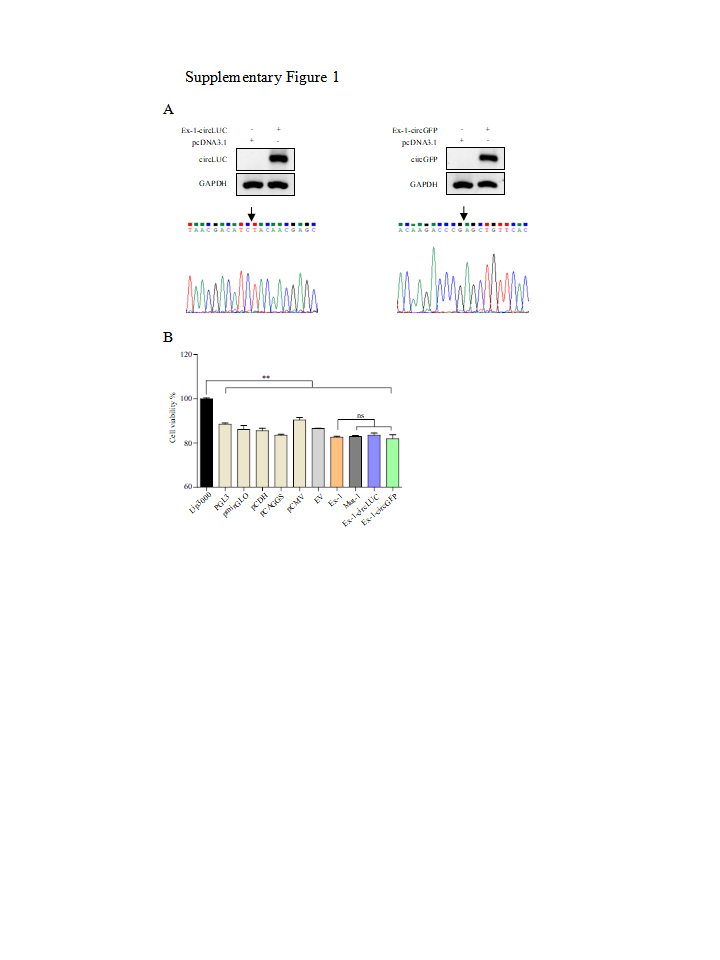

Supplement: FIG S1 [file mbio.02984-21-sf001.tif]

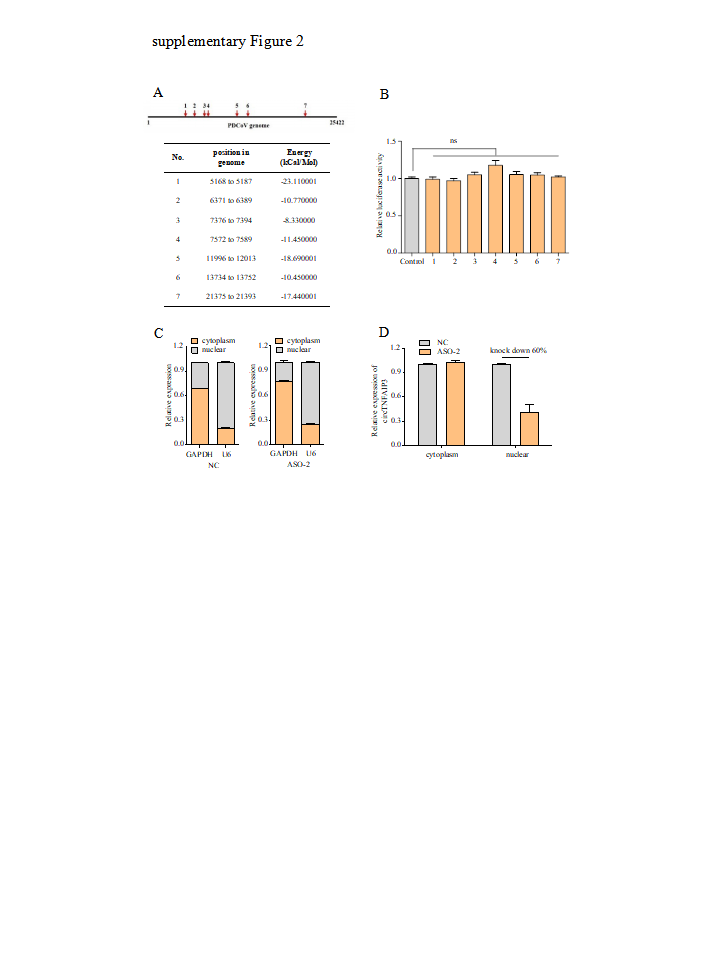

Supplement: FIG S2 [file mbio.02984-21-sf002.tif]
